# Luminescent Tetranuclear Gold(I) Dibenzo[g,p]chrysene Derivatives: Effect of the Environment on Photophysical Properties

**DOI:** 10.3390/molecules25040949

**Published:** 2020-02-20

**Authors:** Francisco J. Caparrós, Mani Outis, Yongsik Jung, Hyeonho Choi, João Carlos Lima, Laura Rodríguez

**Affiliations:** 1 Departament de Química Inorgànica i Orgànica, Secció de Química Inorgànica, Universitat de Barcelona, Martí i Franquès 1-11, 08028 Barcelona, Spain; franciscojavier.caparros@antares.qi.ub.es; 2 Institut de Nanociència i Nanotecnologia (IN2UB). Universitat de Barcelona, 08028 Barcelona, Spain; 3 LAQV-REQUIMTE, Departamento de Química, Universidade Nova de Lisboa, 2829-516 Monte de Caparica, Portugal; m.hosseinzadeh@campus.fct.unl.pt (M.O.); lima@fct.unl.pt (J.C.L.); 4 Samsung Advanced Institute of Technology, Samsung Electronics Co. Ltd., 130 Samsung-ro, Yeongtong-gu, Suwon-si, Gyeonggi-do 16678, Korea; ys327.jung@samsung.com (Y.J.); hono.choi@samsung.com (H.C.)

**Keywords:** gold, dibenzo[g,p]chrysene, heavy atom effect, luminescence matrixes

## Abstract

A new 2,7,10,15-tetraethynyldibenzo[g,p]chrysene ligand (**1**) and two tetranuclear gold(I) derivatives containing PPh_3_ (**3**) and PMe_3_ (**4**) phosphines were synthesized and characterized by ^1^H and ^31^P NMR, IR spectroscopy, and high-resolution mass spectrometry. The compounds were studied in order to analyze the effect of the introduction of gold(I) on the supramolecular aggregation and photophysical properties. Absorption and emission spectra displayed broad bands due to the establishment of π π interactions as an indication of intermolecular contacts and the formation of aggregates. A decrease of the recorded quantum yield (QY) of the gold(I) derivatives was observed compared to the uncomplexed ligand. The introduction of the complexes into poly methyl methacrylate (PMMA) and Zeonex 480R matrixes was analyzed, and an increase of the measured QY of **4** in Zeonex was observed. No phosphorescent emission was detected.

## 1. Introduction

In recent years, increasing attention has been paid to disk-shaped organic molecules such as polycyclic aromatic hydrocarbons (PAHs) because of their characteristic structural and electronic properties for extended π-conjugation [1,2,3,4]. Dibenzo[g,p]chrysene (DBC) derivatives are one of these types of compounds due to their high absorption coefficients, fluorescence quantum yield, and photostability. These properties have led to DBC being regarded as a model compound for the study of photofunctional organic devices: pigments; dyes, and optical photovoltaic applications [5,6,7,8].

To build well designed DBC derivatives, a suitable synthetic model is required, allowing properties to be tuned by chemical modifications at the meta-, para-, or both meta- and para-positions [6,8,9]. Taking the available DBC skeleton into consideration and the established procedure used to obtain the tetrabrominated DBC derivative [9], the aromaticity of a compound can be extended by the introduction of an alkynyl moiety at each brominated position via Sonogashira coupling. This creates a new possibility of the coordination of metallic complexes via the formation of M-C≡C bonds. The linearity of the M-C≡C moiety and the preference of gold(I) for linear coordination makes alkynyl gold(I) compounds [10,11] attractive candidates for the design of discotic molecules derived from a DBC core.

Gold(I) complexes are very well known to display aurophilic (Au(I)···Au(I)) interactions, and this particular property modulates the energy of excited states while retaining the coordination sphere and remaining capable of considerably affecting the photophysical properties of the polymetallic molecules [12]. These aggregates can affect the molecules’ photophysical properties and introduce a scaling up of the spin–orbit coupling (SOC), which is enhanced by metallophilic bonding. Phosphorescence is a distinctive photophysical property of transition-metal complexes, mainly those containing heavy atoms, due to efficient spin–orbit coupling (SOC) that relaxes the spin selection rule, favors triplet state population, and increases the radiative rate for triplet emission. However, ligand-dominated fluorescence is frequently observed in some polyaromatic complexes [13,14,15,16,17]. Hence, it has become clear that the presence of heavy elements does not guarantee a fast intersystem crossing (ISC) rate; the molecular structure and the nature of the ligands, and the relative positions of singlet and triplet states, play a critical role in determining the intersystem crossing (ISC) rate.

In this work, we report the synthesis of a new tetraalkynyl DBC (TADBC) ligand and the formation of two gold(I) derivatives containing a phosphine ligand at the second coordination position, these being, to the best of our knowledge, the first examples of gold(I) alkynyl DBC complexes reported in the literature. The effect of the gold(I) coordination on the photophysical properties of the DBC and the bulkiness of the phosphine, and the impact of the aggregation induced by the additional aurophilic properties on the resulting emission, were herein explored in different media (solution and organic matrixes).

## 2. Results and Discussion

### 2.1. Synthesis and Characterization

**Scheme 1:**
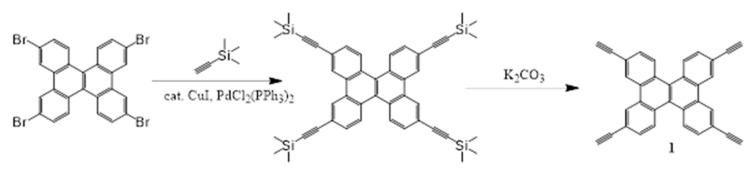
Synthesis of the 2,7,10,15-tetraethynyldibenzo[g,p]chrysene (TADBC) ligand **1**.

**Scheme 2:**
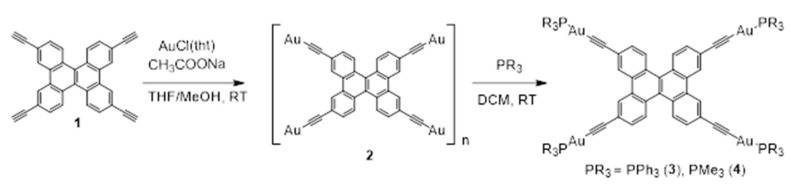
Synthesis of the TADBC gold(I) complexes **3** and **4**.

Two tetranuclear gold compounds containing a 2,7,10,15-tetraethynyldibenzo[g,p]chrysene (**1**) group as a chromophore and two different phosphines (PMe_3_ for compound **3** and PPh_3_ for compound **4**) at the second coordination position were synthesized following the reaction procedures described in Scheme 1 and Scheme 2. Initially, the ligand 2,7,10,15-tetrabromodibenzo[g,p]chrysene that was obtained following the procedure reported in the literature [9], and was reacted with ethynyl-(trimethyl)silane via a Sonogashira coupling with subsequent removal of the TMS-protecting group with potassium carbonate, giving compound **1** in a moderate yield (Scheme 1). ^1^H NMR and IR spectroscopies and mass spectrometry verified the correct formation of the product.

The reaction of **1** with AuCl(tht) (tht = tetrahydrothiophene) in a 1:4 ratio and in the presence of sodium acetate as a base gave rise to the formation of the insoluble polymer **2** [18]. The successful formation of the polymer was evidenced by the disappearance of C≡*C-H* vibration of the terminal alkynyl proton of **1** in IR spectroscopy and the insoluble character of the resulting compound. Next, the reaction of a dichloromethane suspension of the polymer (**2**) with a stoichiometric amount of triphenylphosphane (PPh_3_) or trimethylphosphane (PMe_3_) allowed the formation of the desired products **3** and **4**, respectively, in good yields. Polymer **2** was also reacted with the dppm (PPh_2_CH_2_PPh_2_) in a 1:2 ratio and dppb (PPh_2_(CH_2_)_4_PPh_2_) in a 1:1 or 1:2 ratio, but we were not able to obtain the expected diphosphine products in pure form.

Both gold compounds **3** and **4** were successfully characterized by ^1^H and ^31^P NMR, IR, and mass spectrometry. The ^1^H NMR spectra showed the signals of both the chromophore, slightly upfield shifted due to the Au-PR_3_ coordination, and the phosphine. Additionally, the terminal alkynyl proton signal at 3.28 ppm of the free ligand **1** disappeared providing a direct indication of the formation of the complex. ^31^P{^1^H} NMR spectra showed in all cases only one signal, which was ca. 50 ppm downfield-shifted with respect to the free phosphine, indicating the formation of only one product. The detection of the [M] molecular peak by MALDI-TOF mass spectrometry in all cases, at *m/z* 424.2, 2254.4, and 1512.2 for **1**, **3**, and **4** respectively, was unequivocal evidence of the successful formation of the complexes (see Supplementary Materials). The presence of higher mass peaks identified as M + C≡CAuPR_3_ at 2082.6 in the case of **4** led us to consider that the compound might be stable in the form of aggregates. Several attempts were performed to grow single crystals suitable for X-ray diffraction resolution, but these failed.

### 2.2. Photophysical Characterization

The absorption and emission spectra of both complexes **3** and **4** and of the free ligand **1** were recorded for 1 × 10^−5^ M dichloromethane solutions at room temperature, and the obtained data are summarized in Table 1.

**Figure 1:**
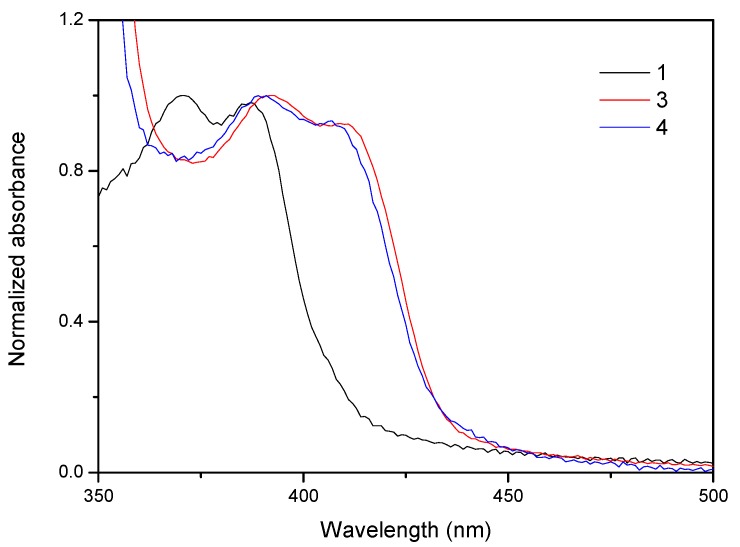
Normalized absorption spectra of 1 × 10^−5^ M solutions of both complexes **3** and **4** and the free ligand **1** in dichloromethane.

The electronic absorption spectra of all the complexes (Figure 1) showed an intense band with vibronic resolution at 380-420 nm. These bands displayed the same profile as the corresponding spectrum of the ligand **1**, but, as is commonly observed [11], they were ca. 20 nm red-shifted due to coordination to the metal atom. The vibranic resolution with spaces of ca. 1300 cm^−1^ indicated an origin in π–π* intraligand transitions (^1^IL) origin that were perturbed by the gold metal atom. The recorded broadening of the spectra in **3** and **4** with respect to **1** was ascribed to π–π interactions between aromatic moieties (intermolecular interactions or adducts) [19].

**Figure 2:**
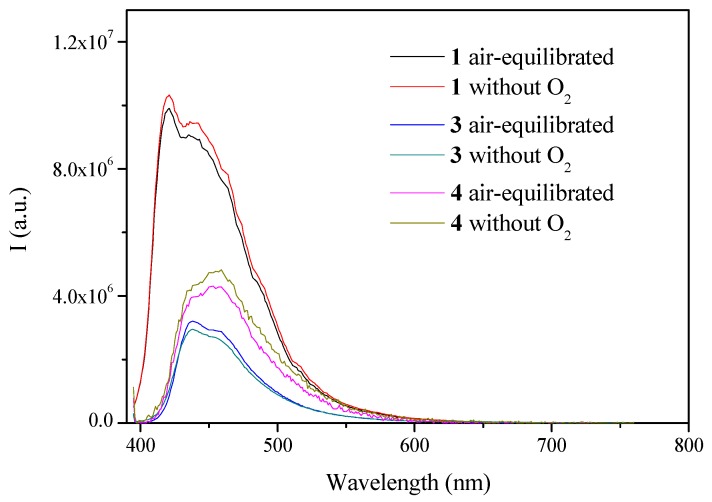
Emission spectra of 1 × 10^−5^ M of the compounds **3** and **4** and of the free ligand **1** in dichloromethane with and without oxygen (λ_exc_ = 345 nm).

Emission spectra were recorded in the presence and in the absence of O_2_ (Figure 2). Fluorescence bands with sharp vibrational modes originating from the TADBC monomer were recorded [20]. A small red-shift (ca. 20 nm) of the emission of the gold complexes with respect to the uncomplexed ligand was observed, and no significant variation of the emission intensity was noted upon removing oxygen. These facts, together with the short decay times associated with the luminescent bands (Table 1), made us confident in assigning them to ^1^IL fluorescence transitions. It was observed that compound **3**, containing the bulkier phosphine, displayed the common shape of TADBC monomer emission of **1**, while **4** displayed a different peak-to-shoulder intensity proportion, which might have been an indication of the presence of aggregates [21,22].

Fluorescence decay times were measured for all the compounds using time-correlated single-photon counting (TC-SPC) (Table 1). The decay times collected at 450 nm were of the order of a few nanoseconds, as expected for fluorescence emissions. A ca. 4-fold decrease of the fluorescence quantum yields was observed upon coordination of the Au-PR_3_ fragment due to population of the (low-energy and non-emissive) triplet state in **3** and **4** (Table 2). These fluorescence quantum yields were not affected by oxygen due to the short lifetimes of the singlets, and were also evidence that reverse intersystem crossing, either thermally activated or due to triplet–triplet annihilation, was not occurring in these systems [23].

**Figure 3:**
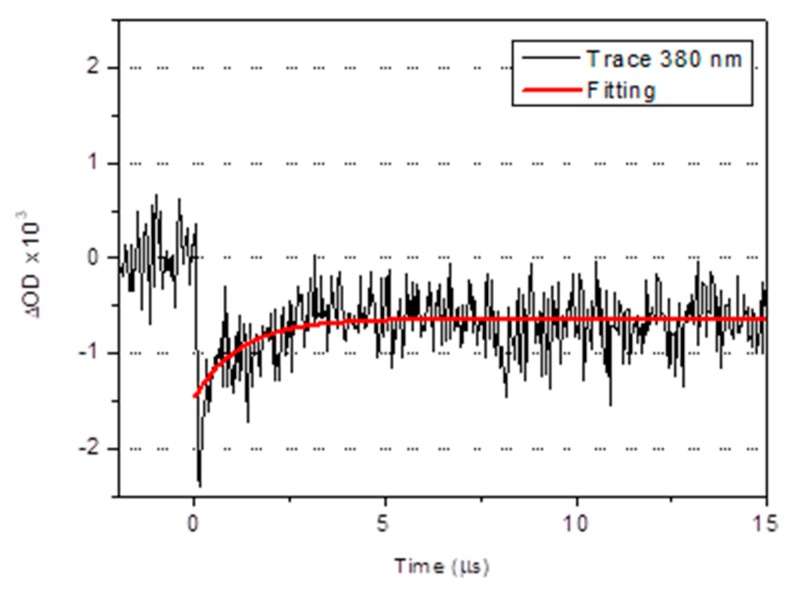
Bleaching and recovery of the transient absorption at 380 nm following excitation with ns pulse of 355 nm for compound **3** in dichloromethane without O_2_.

The presence of a triplet population was further confirmed by transient absorption spectroscopy (nanosecond laser flash photolysis, NLFP) in de-aerated solutions with compound **3**. We observed the presence of transients and the ground state bleaching recovered with a risetime of ca. 1 μs, in agreement with a populated triplet state with fast deactivation through non-radiative relaxation pathways (Figure 3).

**Figure 4:**
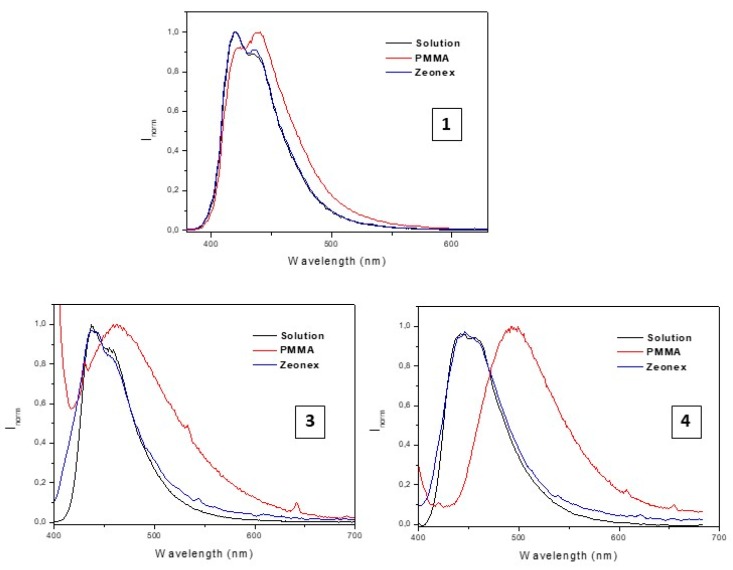
Normalized emission spectra of **1**, **3**, and **4** in 10^−5^M dichloromethane solution and in solid matrixes of PPMA and Zeonex.

In order to check the viability of blocking non-radiative pathways for the decay of the triplet state, and to obtain gold(I) luminescent materials (films), the compounds were dispersed in solid matrixes of PMMA and a cyclic olefin copolymer, Zeonex (Figure S12), and the corresponding emissions in these media was measured (Figure 4). In all cases, a low-lying triplet emission was not detected. A red-shift of the emission band in the more polar matrix, PMMA, was observed. This red-shift was more pronounced in the case of the gold derivative **4**, containing the phosphine with less steric hindrance. Additionally, a change of the shape of the emission spectra was observed in PMMA, being completely broad in the case of the gold complexes (Figure 4). This was compatible with excimer emission, which could be ascribed to the higher polarity of the PMMA matrix and the highly apolar character of the gold complexes, allowing them to self-assemble in this medium. The larger affected changes in **3** and **4** with respect to **1** supported the involvement of the Au(I) atom in the Au···Au or Au···π excimeric aggregates [7,24,25].

**Figure 5:**
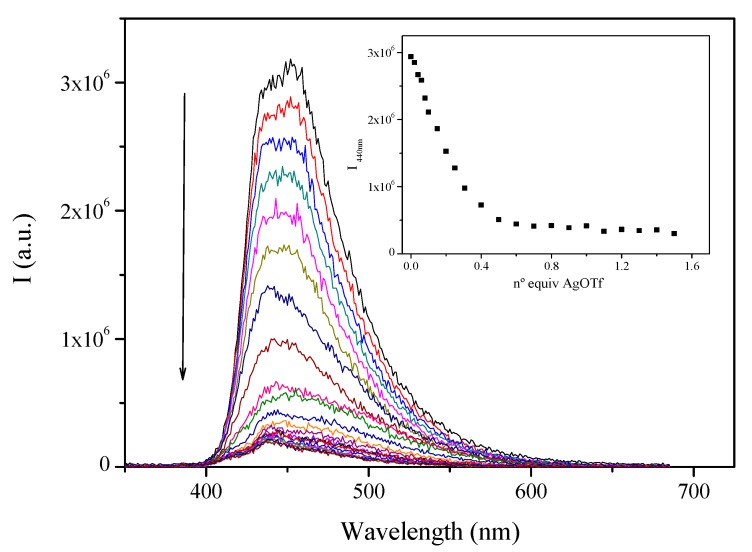
Emission spectra of a 1 × 10^−5^M dichloromethane solution of **4** upon addition of increasing amounts of AgOTf. Inset: variation of emission maxima at 440 nm at each titration point.

The presence of alkynyl and aromatic moieties in the studied complexes led us to the possibility of obtaining heterometallic complexes containing not only Au(I), but also another heavy metal atom, such as Ag(I), able to coordinate to these groups. In order to ascertain the adequate stoichiometry, dichloromethane solutions of **3** and **4** were titrated with silver triflate salt and the resulting emission changes were followed at each point. The plot of the emission maxima against equivalents of Ag^+^ suggested the formation of a 1:1 complex in the case of **3**, containing a bulkier phosphine, and 1:0.5 in the case of **4**, with the PMe_3_ phosphine (Figure 5).

**Figure 6:**
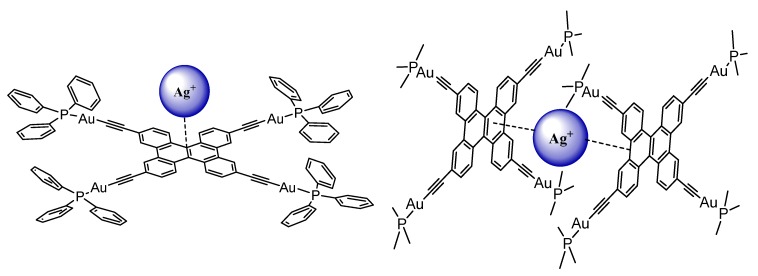
Schematic representation of the expected heterometallic complexes derived from **3** and **4** with AgOTf.

The different stoichiometry of the resulting heterometallic complexes may have been attributed to the bulkiness of the PPh_3_ phosphine in **3** with respect to PMe_3_ in **4**, as exemplified in Figure 6.

The formation of these structures was supported by ^1^H and ^31^P NMR analysis. The aromatic protons of the DBC became completely broader and were not possible to identify by ^1^H NMR due to the Ag···π coordination. This fact has been previously observed in Au···π aggregates [26]. ^31^P NMR was slightly downfield shifted (3-6 ppm) since the phosphine moiety was not directly affected by the coordination of the silver salt (Figures S13–S16). The changes in the absorption profile also supported the formation of these heterometallic products where the band became clearly broader after coordination to the Ag^+^ cation (Figure S17).

A ca. 20% increase in the resulting quantum yield of the heterometallic complex with **4** (a less bulky compound) was observed when the system was introduced into Zeonex (see Table 2). This value was very close to those previously recorded for the homometallic complexes both in solution and within thin films and, thus, we were able to obtain, for the first time, a luminescent material containing the heterometallic Au-Ag DBC structure.

## 3. Conclusions

The synthesis of a tetraalkynyl derivative from 2,7,10,15-tetrabromodibenzo[g,p]chrysene was performed via Sonogashira coupling, allowing us to obtain uncommon tetranuclear DBC derivatives.

Absorption and emission data showed that the photophysical properties of all compounds were driven by the ππ ^1^IL transitions of the organic ligand. Population of the triplet state in the presence of gold(I) atoms was detected by laser flash photolysis experiments, but the corresponding phosphorescence emission could not be detected.

The introduction of the compounds into a PMMA matrix led to a shift of the resulting emission color from blue to greenish due to the presence of aggregates. Additionally, in the case of the less bulky gold compound, the presence of a second metal atom (silver) in the structure induced a ca. 20% increase of the measured quantum yields when the heterometallic system was introduced into Zeonex.

## 4. Experimental Section

### 4.1. General Procedures

All manipulations were performed under prepurified N_2_ using standard Schlenk techniques. All solvents were distilled from appropriated drying agents. Commercial reagents 1,1,2,2-tetraphenylethylene, bromine, 2,3-Dichloro-5,6-dicyano-1,4-benzoquinone, trifluoromethanesulfonic acid, ethynyltrimethylsilane, copper iodine, PPh_3_, and PMe_3_ were purchased from Aldrich, and potassium carbonate and sodium acetate were purchased from Panreac. The synthesis of 2,7,10,15-tetrabromodibenzo[g,p]chrysene was carried out following the procedure described in the literature [9].

### 4.2. Physical Measurements

Infrared spectra were recorded on a FT-IR 520 Nicolet. ^1^H NMR (δ(TMS) = 0.0 ppm) and ^31^P{^1^H}-NMR (δ(85% H_3_PO_4_) = 0.0 ppm) spectra were obtained on a Varian Mercury 400 and Bruker 400 A 4800Plus Matrix-Assisted Laser Desorption/Ionization MALDI TOF/TOF Analyzer Applied Biosystems MDS SCIEX was used to record mass spectra. Absorption spectra were recorded on a Varian Cary 100 Bio UV spectrophotometer and emission spectra on a Horiba-Jobin-Ybon SPEX Nanolog spectrofluorimeter. Luminescent quantum yields were recorded using an Absolute PL quantum yield spectrometer from Hamamatsu Photonics upon excitation the samples at 345 nm. Absorption transient spectra were measured with a laser flash photolysis LK60 Applied Photophysics system in emission mode, collecting the absorption recovery at 380 nm after excitation with ns pulse laser pulse at 355 nm. Fluorescence lifetimes (*τ*) were measured via the time-correlated single-photon counting technique (TCSPC) using home-built equipment upon excitation of the samples at 370 nm. The lamp pulses were monitored using a synchronization photomultiplier; the PM signal was shaped in a constant fraction discriminator (Canberra 2126) and directed to a time-to-amplitude converter (TAC, Canberra 2145) as start pulses. Emission wavelength was selected with a monochromator (Oriel 77250) imaged in a fast photomultiplier (9814B Electron Tubes Inc.) and the PM signal was shaped as described above, and delayed before entering the TAC as stop pulses. The analogue TAC signals were digitized (ADC, ND582) and stored on a PC. Decays were analyzed by a Python 2.7 written Software developed by M. Outis using the reconvolution method with inclusion of background noise and color shift parameters. The goodness of fit was checked by chi-squared value and quality of weighted residuals and autocorrelation function.

### 4.3. Synthesis of the Compounds

#### 4.3.1. Synthesis of **1**

First, 2,7,10,15-tetrabromodibenzo[g,p]chrysene (600 mg, 0.93 mmol) was dissolved in 50 mL of diethylamine. A mixture of CuI (4.5 mg, 0.5%mol), PdCl_2_(PPh_3_)_2_ (32.7 mg, 1.2%mol), and ethynyltrimethylsilane (91.5 mg, 0.93 mmol) was then added to the solution. After 24 h stirring at 50 °C, the solvent was removed under reduced pressure and the product was purified by flash chromatography on a silica gel column with CH_2_Cl_2_ as an eluent. The resulting product was dissolved in 60 mL of MeOH/CH_2_Cl_2_ (*v*/*v* = 2:1), after which 211.4 mg of K_2_CO_3_ (2.79 mmol) was added to the solution and the mixture was stirred for 2h. After this time, the solvent was removed under vacuum. The resultant solid was dispersed in 20 mL of CH_2_Cl_2_ and filtered. The solvent was removed under vacuum to obtain the product as an orange solid, yielding 150 mg of pure compound (yield = 25%). ^1^H NMR (400 MHz, CDCl_3_): δ 3.28 (s, 4H), 7.74 (d, *J* = 8.5 Hz, 4H), 8.54 (d, *J* = 8.6, 4H), 8.81 (s, 4H). MALDI-TOF MS(+) *m/z* 424.2 ([1]^+^, calcd *m/z* 424.2), 400.2 ([**1** -C≡C-H + H]^+^, calcd 399.2). ATR-FTIR (cm^−1^): 1436 (C=C), 2103 (C≡C), 3049 (C_sp2_-H), 3289 (C≡C-H). Anal. found (calcd for C_34_H_16_): C, 96.28 (96.20); H, 3.73 (3.80).

#### 4.3.2. Synthesis of **2**

A solution of **1** (100 mg, 0.24 mmol) in 20 mL THF/MeOH (*v*/*v* =1:1) was added to a mixture of AuCl(tht) (302 mg, 0.96 mmol) and sodium acetate (154.6 mg, 1.92 mmol). An orange precipitate appeared immediately. The solution was stirred for 1 h. The solid was isolated after filtration under vacuum and washing with THF and methanol, yielding 244.8 mg of **2** (yield = 86%). ATR-FTIR (cm^−1^): 1560 (C=C), 2096 (C≡C), 2950 (C_sp2_-H). Anal. found (calcd for C34H_12_Au_4_): C, 33.86 (33.80); H, 1.04 (1.00).

#### 4.3.3. Synthesis of **3**

A solution of PPh_3_ (17.3 mg, 0.066 mmol) in 20 mL of CH_2_Cl_2_ was added to a suspension of **2** (20 mg, 0.017 mmol) in the same solvent (10 mL). The mixture was stirred for 1 h and the complete solubilization of the polymer **2** was observed. The resulting solution was reduced until it reached half of the initial volume and the product was precipitated by the addition of hexane. The product was recrystallized with CH_2_Cl_2_/hexane, yielding 19.2 mg of **3** (yield = 50%). ^1^H NMR (400 MHz, CDCl_3_): δ 7.44-7.64 (m, 60H), 7.70 (d, *J* = 9.2 Hz, 4H), 8.49 (d, *J* = 8.5, 4H), 8.82 (s, 4H). ^31^P{^1^H} NMR (161.9 MHz CHCl_3_, ppm): δ 42.2 (s). MALDI-TOF MS(+) *m/z*: 2257.4 ([**3** + H]^+^, calcd 2257.4), 1799.3 ([**3** -AuPPh_3_ +H]^+^, calcd 1799.5), 1340.3 ([**3** -2AuPPh_3_ +2H]^+^, calcd 1340.2). ATR-FTIR (cm^−1^): 1574 (C=C) 2100 (C≡C), 3043 (C_sp2_-H). Anal. found (calcd for C_106_H_72_Au_4_P_4_): C, 56.45 (56.40); H, 3.27 (3.21).

#### 4.3.4. Synthesis of **4**

The synthesis of complex **4** was performed following the same procedure as **3** with substitution of the phosphine PPh_3_ for PMe_3_ (66 µL of a commercial solution 1 M in THF, 0.066 mmol). Yield = 45% (11.6 mg). ^1^H NMR (400 MHz, CDCl_3_): δ 1.56 (d, *J* = 10.1 Hz, 36H), 7.66 (d, *J* = 8.7 Hz, 4H), 8.45 (d, *J* = 8.5 Hz, 4H), 8.76 (s, 4H). ^31^P{^1^H} NMR (161.9 MHz CHCl_3_, ppm): δ 1.2 (s). MALDI-TOF Ms(+) *m/z* 1512.2 ([3]^+^, calcd *m/z* 1512.2), 1240.2 ([**3** -AuPMe_3_ +H]^+^, calcd *m/z* 1240.2), 968.2 ([**3** -2AuPMe_3_ +2H]^+^, calcd *m/z* 968.2). ATR-FTIR (cm^−1^): 1558 (C=C) 2093 (C≡C), 2962 (C_sp2_-H). Anal. found (calcd for C_48_H_48_Au_4_P_4_): C, 36.53 (36.60); H, 3.24 (3.20).

#### 4.3.5. Preparation of Matrixes Including Compounds

PMMA and Zeonex (Zeon Corporation, Japan) were used as matrix polymers. The films were prepared via drop-casting, using a mixture in chloroform of dopant and host (Zeonex/PMMA). A 100 µL solution of 200 mg/mL of polymer was added to the same volume of a solution of the sample at a concentration of 20 µg/mL. The films were drop-cast onto a quartz substrate at room temperature to avoid any thermal annealing.

## Figures and Tables

**Table 1:** Absorption and emission data of free ligand **1** and the compounds **3** and **4** in dichloromethane at 1 × 10^−5^ M. (λ_exc_ = 345 nm).

Compound	Absorption λ_max_ (nm) (10^4^ ε (M^−1^ cm^−1^)	Emission (Solution, λ_max_ (nm))	τ
**1**	371 (2.0), 388 (1.9)	424, 442	3.65 ns
**3**	390 (5.4), 411 (5.0)	444, 460	3.90 ns
**4**	393 (3.3), 413 (3.0)	444, 460	1.22 ns

**Table 2:** Quantum yield values of **1**, **3**, and **4** in solution and in solid matrixes of poly methyl methacrylate (PMMA) and Zeonex.

Compound	Solution		PMMA		Zeonex	
	Air	N_2_	Air	N_2_	Air	N_2_
**1**	0.22	0.27	0.20	0.20	0.22	0.22
**3**	0.05	0.05	0.06	0.06	0.07	0.07
**4**	0.06	0.07	0.07	0.07	0.03	0.04
**3·AgOTf**	0.007	0.007	0.012	0.015	0.04	0.04
**4·AgOTf**	0.03	0.03	0.05	0.06	0.05	0.05

## Supplementary Materials

The following are available online, Characterization data of the complexes (1H, 31P NMR and mass spectra). Absorption and emission titrations with AgOTf.
