# Supplementary material for: Luminescent Tetranuclear Gold(I) Dibenzo[g,p]chrysene Derivatives: Effect of the Environment on Photophysical Properties
Source: Molecules. 2020 Feb 20;25(4):949. doi: 10.3390/molecules25040949 (PMC7071073; doi:10.3390/molecules25040949)
Supplement: Supplementary file 1 [file molecules-25-00949-s001.pdf]

Luminescent tetranuclear gold(I) dibenzo[g,p]chrysene derivatives. Effect of the environment on the photophysical properties

Francisco J. Caparrós[a,b], Mani Outis[c], Yongsik Jung[d], Hyeonho Choi,[d] João Carlos Lima[c], Laura Rodríguez[a,b,*]

[a] *Departament de Química Inorgànica i Orgànica. Secció de Química Inorgànica. Universitat de Barcelona, Martí i Franquès 1-11, 08028 Barcelona, Spain. e-mail: laura.rodriguez@qi.ub.es*

[b] *Institut de Nanociència i Nanotecnologia (IN2UB). Universitat de Barcelona, 08028 Barcelona (Spain)*

[c] *LAQV-REQUIMTE, Departamento de Química, Universidade Nova de Lisboa, Monte de Caparica*

[d] *Samsung Advanced Institute of Technology, Samsung Electronics Co. Ltd., 130 Samsung-ro, Yeongtong-gu, Suwon-si, Gyeonggi-do, 16678, Republic of Korea*

Supporting information

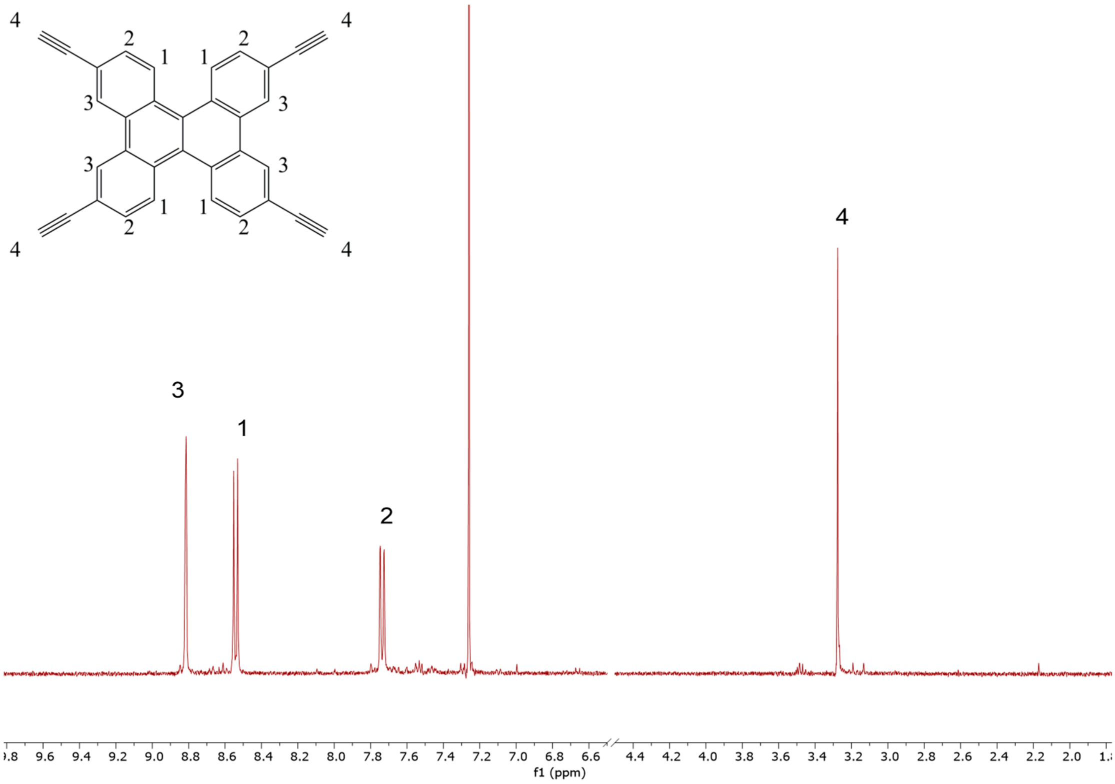

Figure S1. ^{1}H NMR spectrum of **1** in $CDCl_3$.

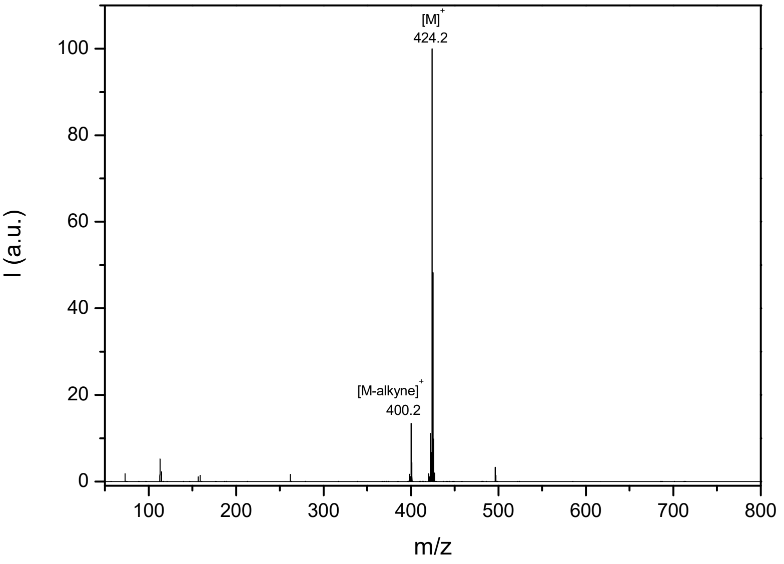

Figure S2. MALDI-TOF Ms(+) spectrum of **1.**

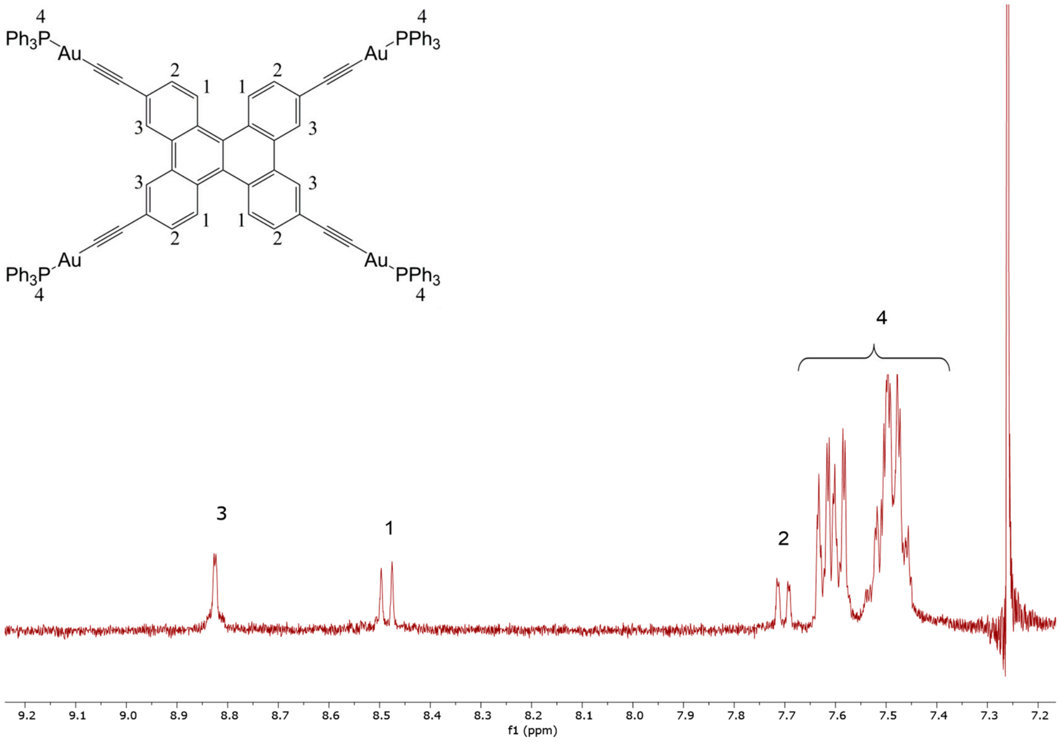

Figure S3. ^{1}H NMR spectrum of **3** in $CDCl_3$.

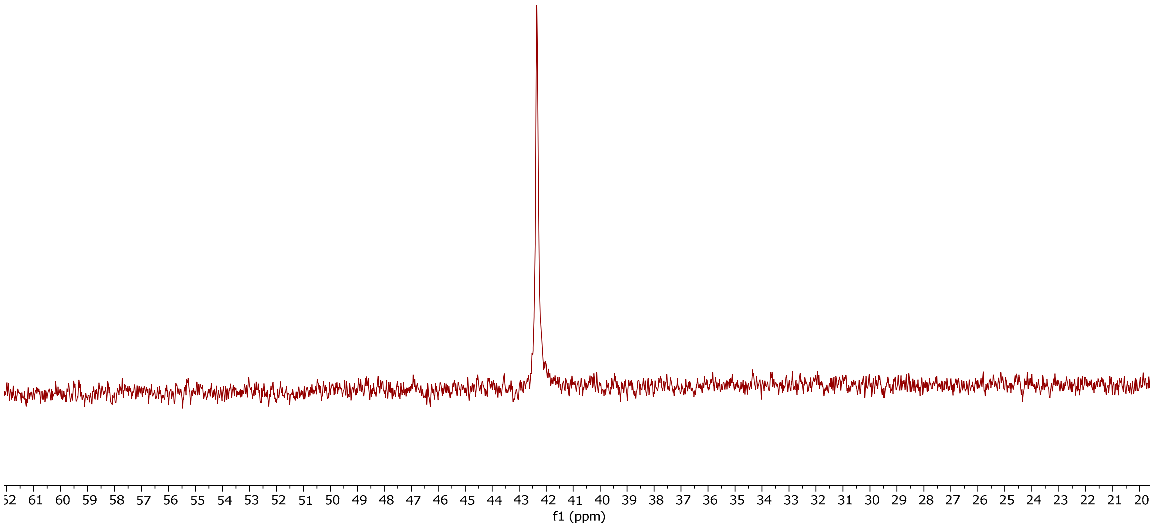

Figure S4. ^{31}P NMR spectrum of **3** in $CDCl_3$.

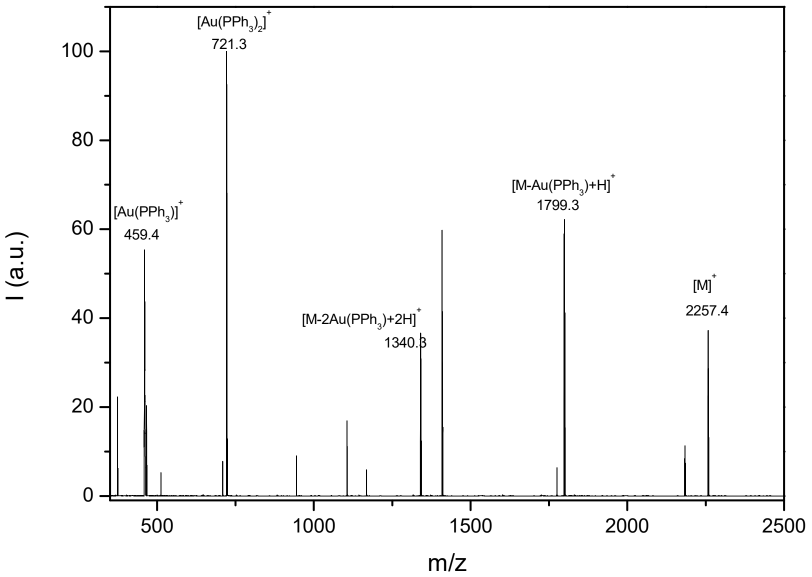

Figure S5. MALDI-TOF Ms(+) spectrum of **3.**

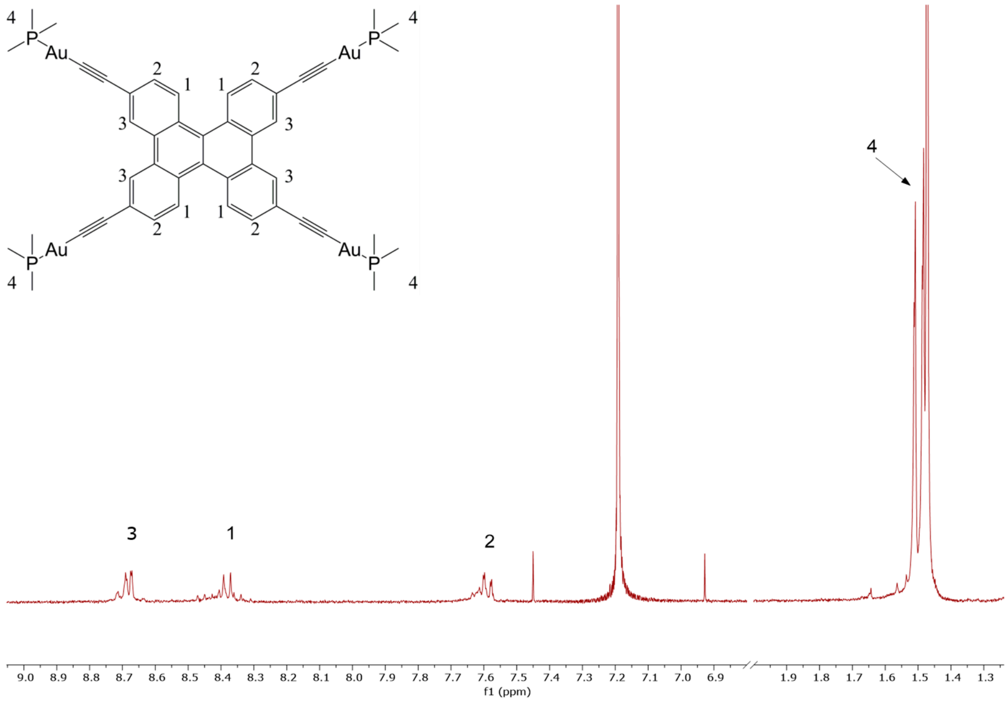

Figure S6. ^{1}H NMR spectrum of **4** in $CDCl_3$.

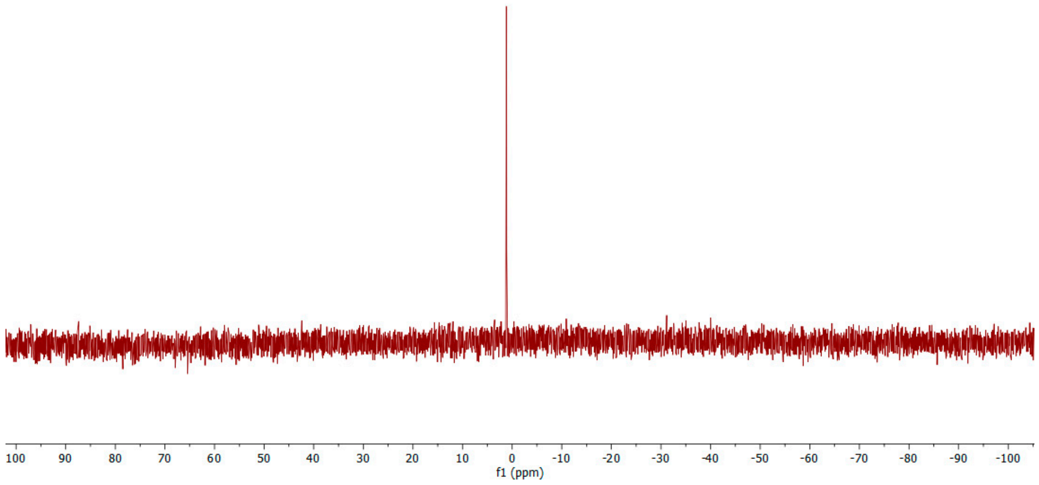

Figure S7. ^{31}P NMR spectrum of **4** in CDCl3.

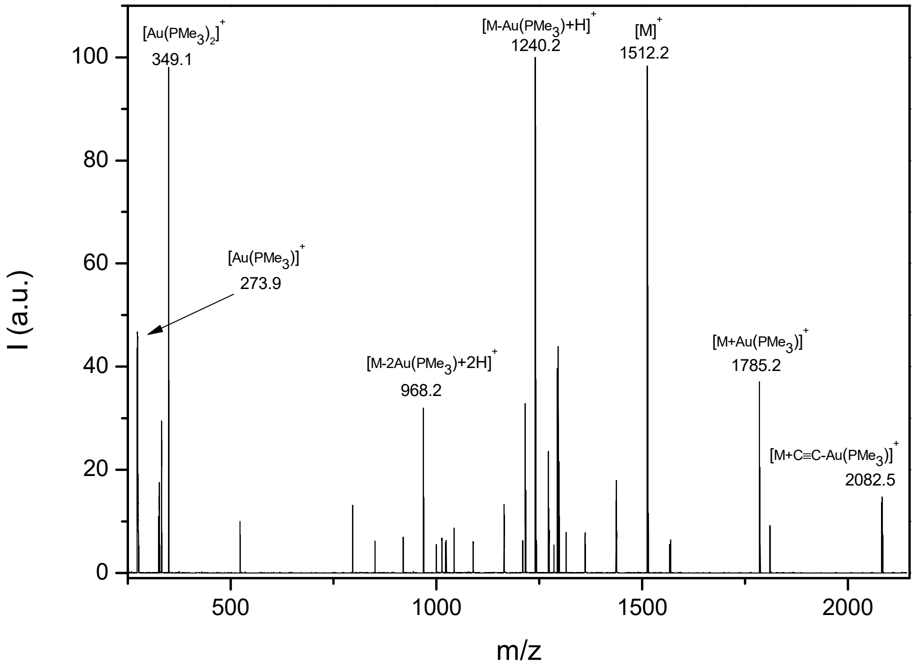

Figure S8. MALDI-TOF Ms(+) spectrum of **4.**

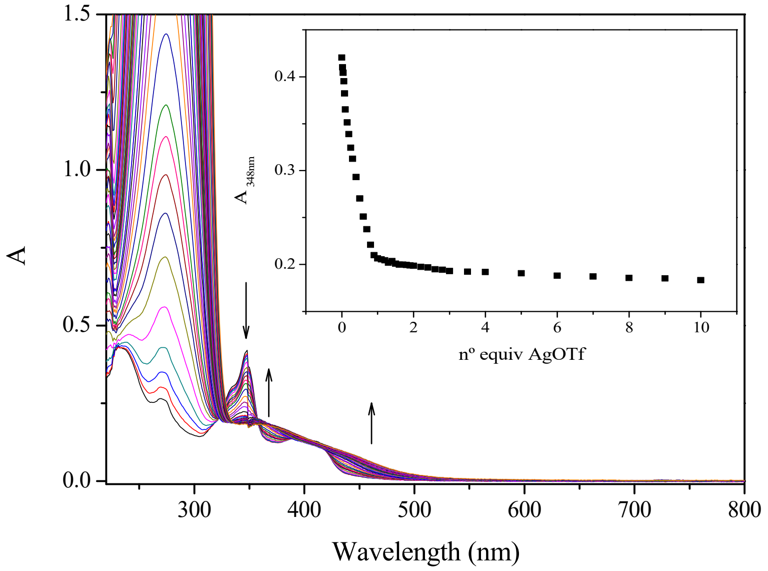

Figure S9. Absorption spectra of 1·10^{-5}M dichloromethane solution of **3** upon addition of increasing amounts of AgOTf. Inset: variation of absorption maxima at 348 nm each titration point.

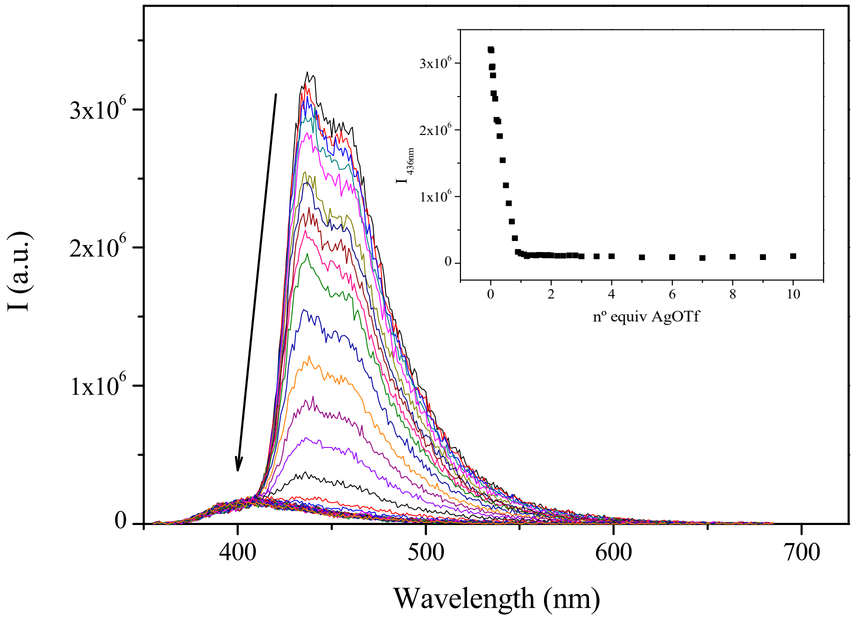

Figure S10. Emission spectra of 1·10^{-5}M dichloromethane solution of **3** upon addition of increasing amounts of AgOTf. Inset: variation of emission maxima at 436 nm at each titration point.

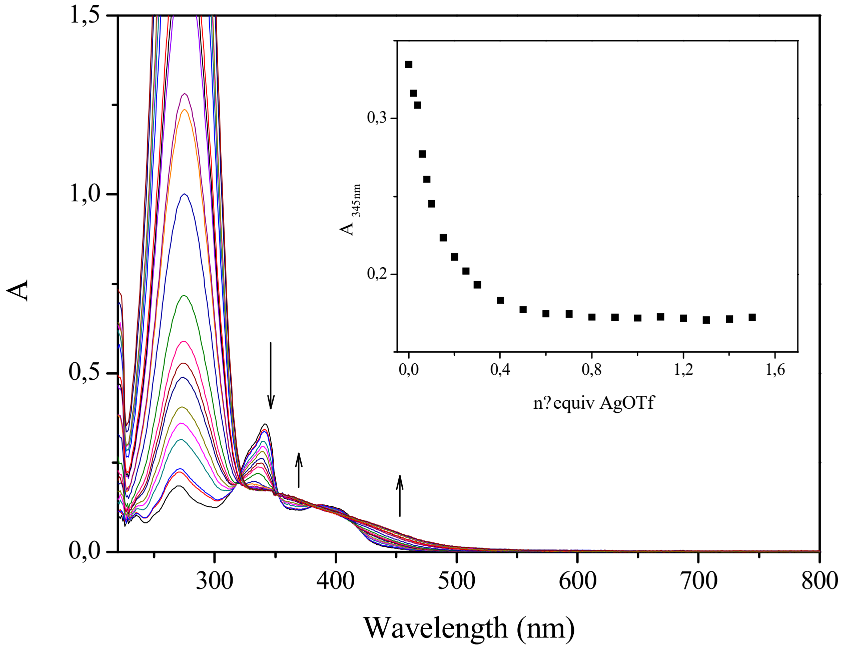

Figure S11. Absorption spectra of $1 \cdot 10^{-5}$M dichloromethane solution of **4** upon addition of increasing amounts of AgOTf. Inset: variation of absorption maxima at 345 nm each titration point.

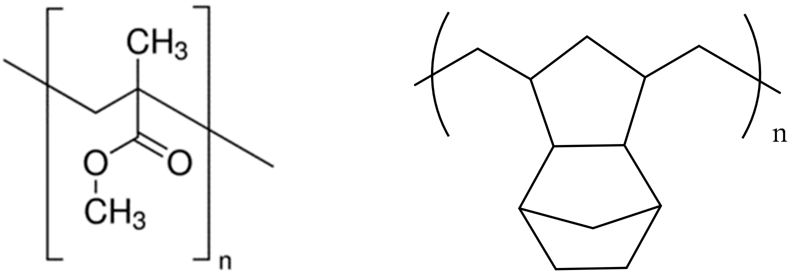

Figure S12. Chemical structure of PMMA, with MW = 996.00 kDa (left) and Zeonex 480R, with MW = 480 kDa (right).

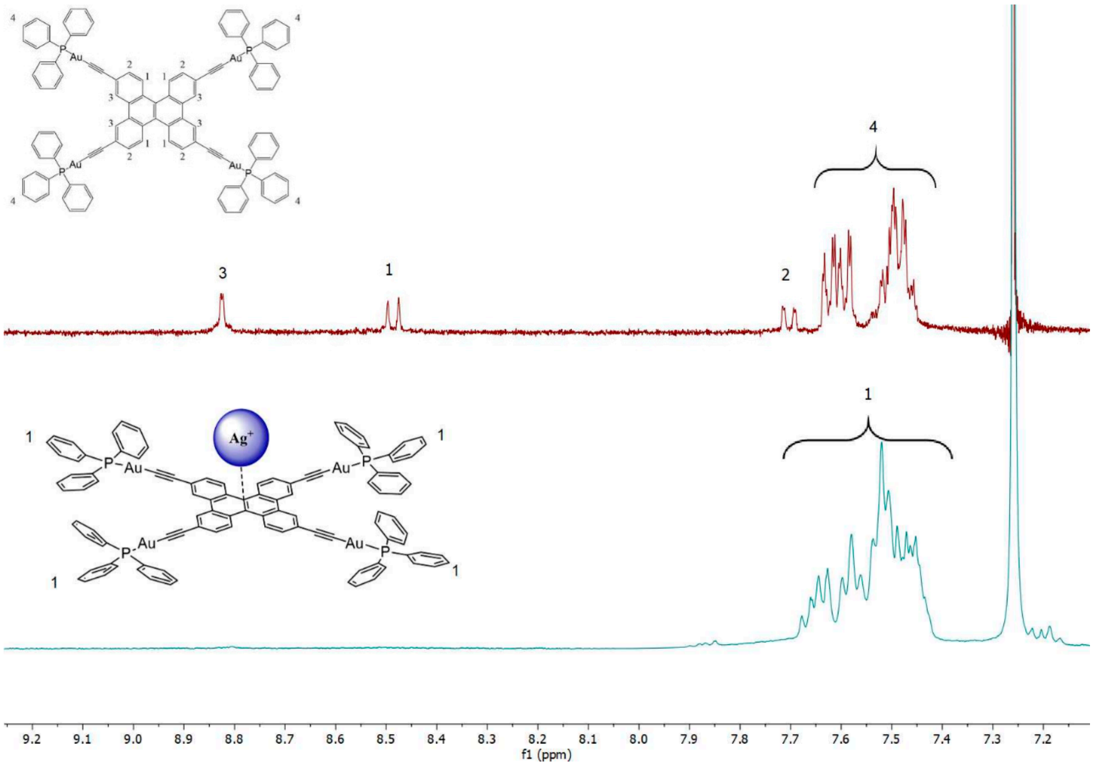

Figure S13. ^{1}H NMR spectra **3** in the presence of 1 equivalent of AgOTf.

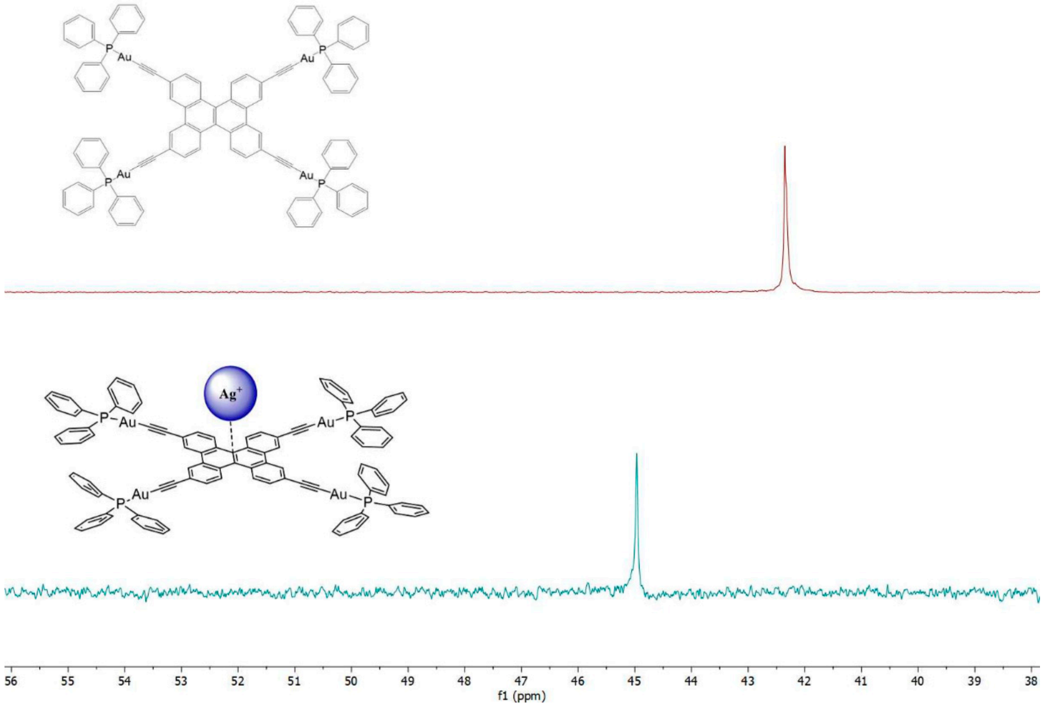

Figure S14. ^{31}P NMR spectra **3** in the presence of 1 equivalent of AgOTf.

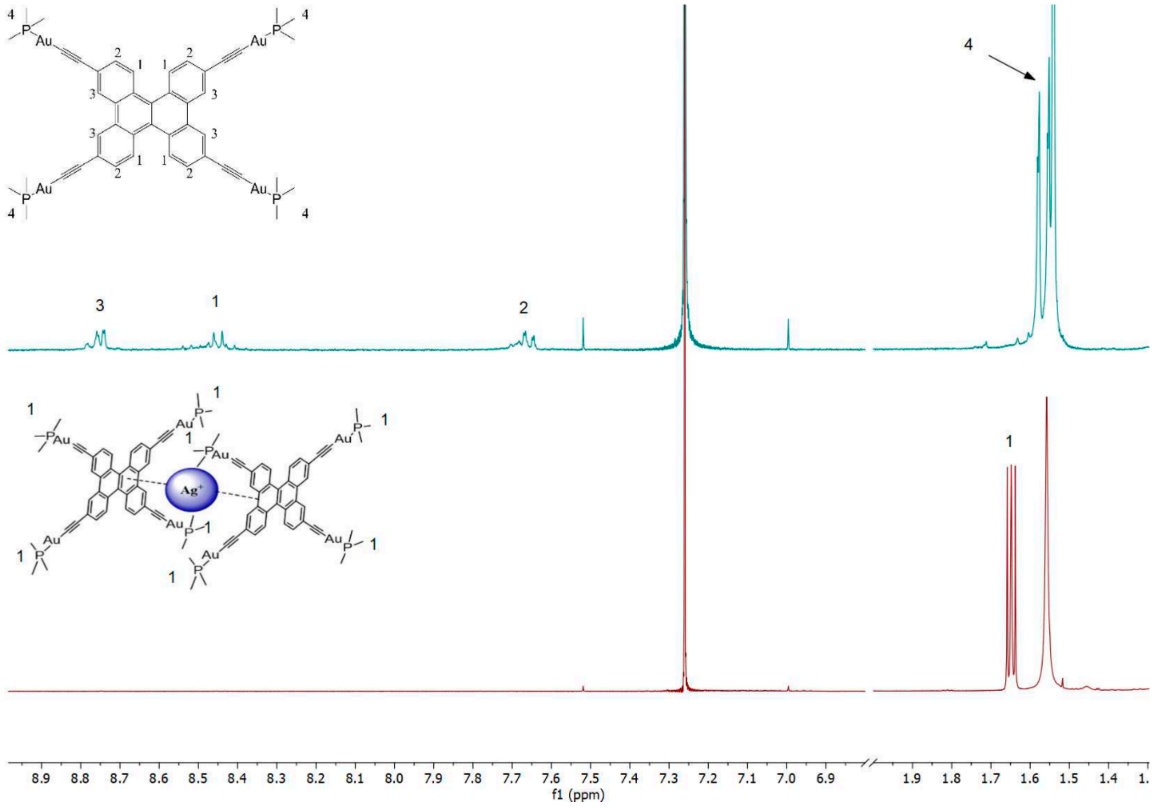

Figure S15. ^{1}H NMR spectra **4** in the presence of 0.5 equivalents of AgOTf.

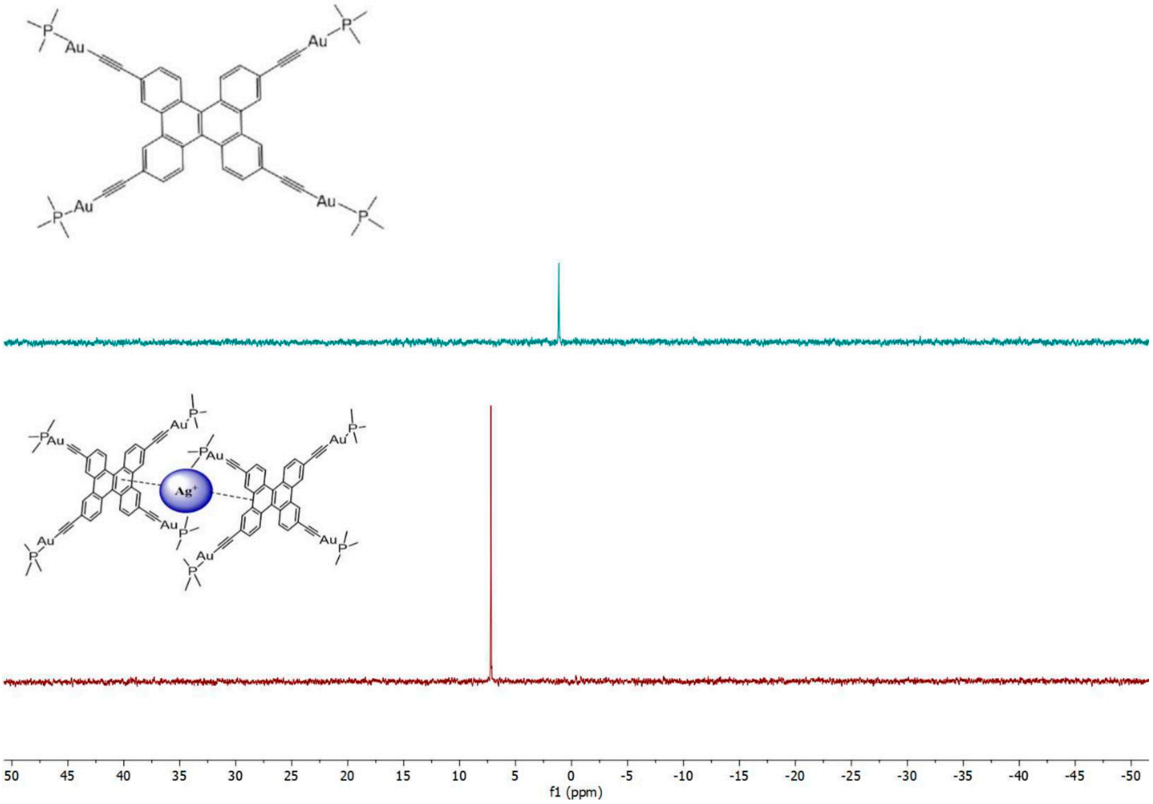

Figure S16. ^{31}P NMR spectra **4** in the presence of 0.5 equivalents of AgOTf.

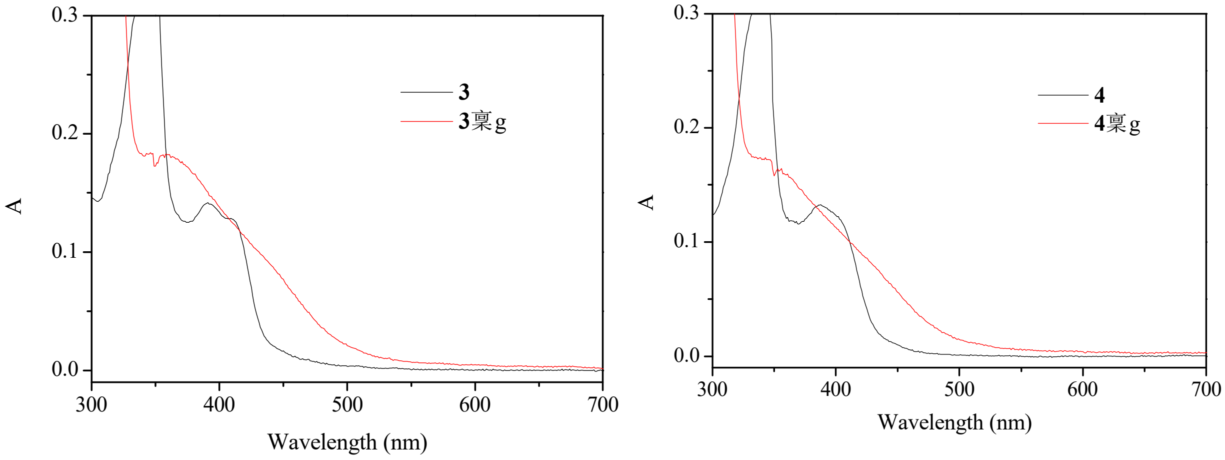

Figure S17. Absorption spectra of **3** and **4** compared with the respective spectra of their Au·Ag heterometallic derivatives

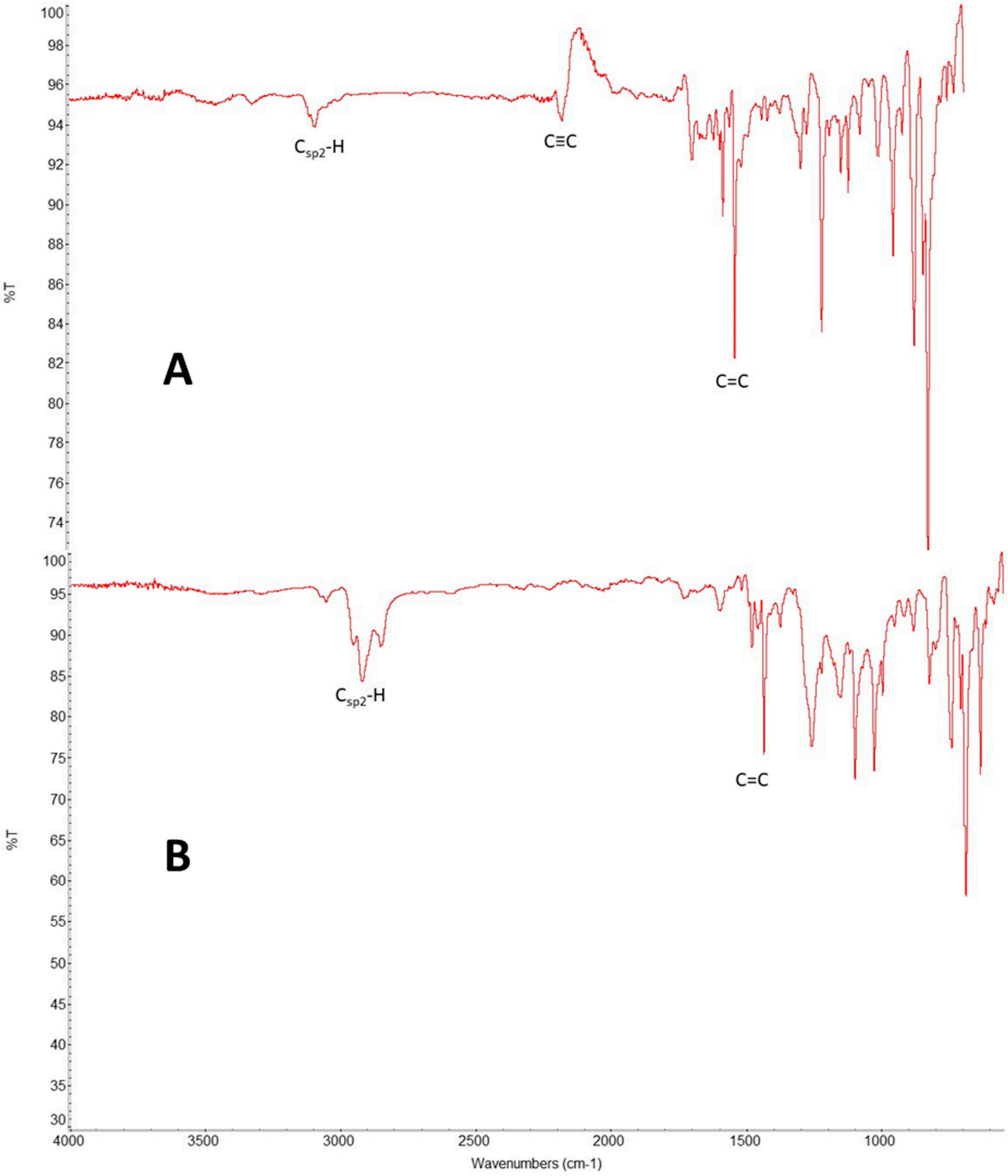

Figure S18. IR spectra of **3** (A) and **3**·Ag (B).

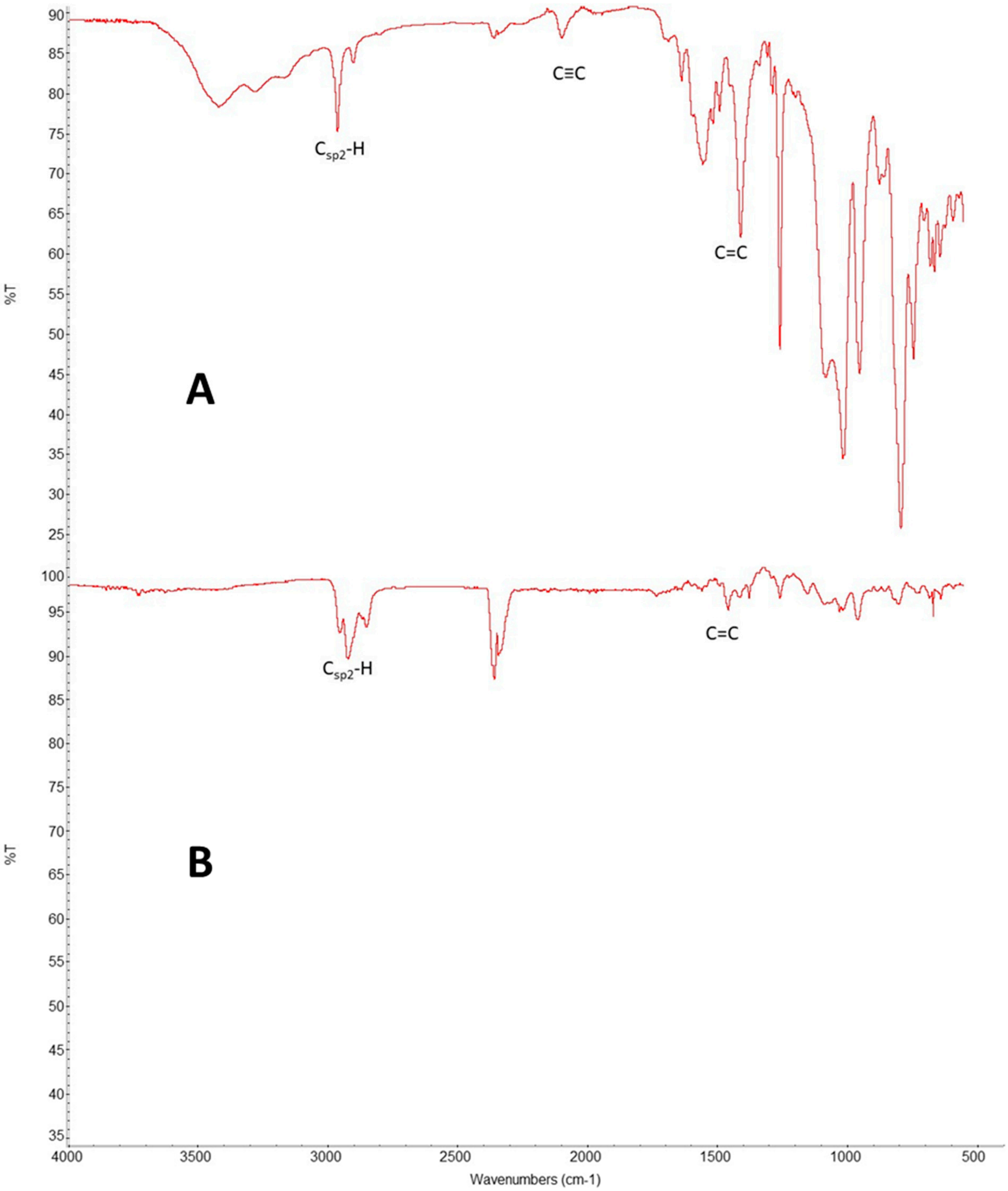

Figure S19. IR spectra of **4** (A) and **4**·Ag (B).
